# Therapeutic potential of oleic acid supplementation in myotonic dystrophy muscle cell models

**DOI:** 10.1186/s40659-024-00496-z

**Published:** 2024-05-17

**Authors:** Nerea Moreno, Maria Sabater-Arcis, Teresa Sevilla, Manuel Perez Alonso, Jessica Ohana, Ariadna Bargiela, Ruben Artero

**Affiliations:** 1 Human Translational Genomics Group, University Institute for Biotechnology and Biomedicine (BIOTECMED), University of Valencia, Valencia, Spain; 2 INCLIVA Biomedical Research Institute, Valencia, Spain; 3 CIBERER, IISCIII, Madrid, Spain; 4 Neuromuscular and Ataxias Research Group, Health Research Institute Hospital, La Fe (IIS La Fe), Valencia, Spain; 5 Department of Medicine, University of Valencia, Valencia, Spain; 6 Centre de Recherche en Myologie, Sorbonne Université, Inserm, Institut de Myologie, Paris, F-75013 France

**Keywords:** Oleic acid, Fatty acid, SCD1, Myoblast differentiation, miR-7, MSI2

## Abstract

**Background:**

We recently reported that upregulation of Musashi 2 (MSI2) protein in the rare neuromuscular disease myotonic dystrophy type 1 contributes to the hyperactivation of the muscle catabolic processes autophagy and UPS through a reduction in miR-7 levels. Because oleic acid (OA) is a known allosteric regulator of MSI2 activity in the biogenesis of miR-7, here we sought to evaluate endogenous levels of this fatty acid and its therapeutic potential in rescuing cell differentiation phenotypes in vitro. In this work, four muscle cell lines derived from DM1 patients were treated with OA for 24 h, and autophagy and muscle differentiation parameters were analyzed.

**Results:**

We demonstrate a reduction of OA levels in different cell models of the disease. OA supplementation rescued disease-related phenotypes such as fusion index, myotube diameter, and repressed autophagy. This involved inhibiting MSI2 regulation of direct molecular target miR-7 since OA isoschizomer, elaidic acid (EA) could not cause the same rescues. Reduction of OA levels seems to stem from impaired biogenesis since levels of the enzyme stearoyl-CoA desaturase 1 (SCD1), responsible for converting stearic acid to oleic acid, are decreased in DM1 and correlate with OA amounts.

**Conclusions:**

For the first time in DM1, we describe a fatty acid metabolism impairment that originated, at least in part, from a decrease in SCD1. Because OA allosterically inhibits MSI2 binding to molecular targets, reduced OA levels synergize with the overexpression of MSI2 and contribute to the MSI2 > miR-7 > autophagy axis that we proposed to explain the muscle atrophy phenotype.

**Supplementary Information:**

The online version contains supplementary material available at 10.1186/s40659-024-00496-z.

## Background

Myotonic dystrophy type 1 (DM1) is a degenerative rare genetic disease with a prevalence of 1/2100 [1]. Most symptoms are neuromuscular, including muscle weakness (myopathy), muscle stiffness and trouble relaxing muscles (myotonia), and progressive muscle wasting (atrophy), but DM1 is characteristically multisystemic and also affects the heart, the brain, and the smooth musculature [2, 3]. DM1 is caused by the pathological expansion of the CTG trinucleotide in the 3´-UTR of the *DM1 protein kinase* (*DMPK*) gene. The pathogenicity is linked to the mutant transcripts that form insoluble hairpins with U-U mismatches, named ribonuclear foci, that sequester RNA-binding proteins of the Muscleblind-like family (MBNL) [4]. These proteins regulate mRNA metabolism, including alternative splicing and polyadenylation [5, 6].

Different pathways have been described that contribute to muscle atrophy in DM1, such as AKT-GSK3β [7, 8], TWEAK/Fn14 [9], AMPK/mTORC1 [10, 11], and PKC [12, 13]. However, in recent years, increased autophagy has gained prominence as an alteration contributing to muscle wasting [14–18]. Pathological activation of autophagy and apoptosis was proved in DM1 myotubes and a *Drosophila* model and was linked to the DM1 atrophic phenotype [14, 18]. Additionally, miR-7 was reported as a critical autophagy repressor by direct translational silencing of critical autophagy genes, namely *ATG7*, *ULK2*, and *ATG4A* [19]. Different works demonstrated that miR-7 was downregulated in DM1 model samples and patient muscle biopsies. Inhibition of miR-7 activity in control myotubes induced atrophic phenotypes similar to those observed in DM1. In contrast, supplementation of miR-7 by a mimetic in DM1 muscle cells restored basal levels of autophagy and improve muscle atrophy and cell differentiation capacity [16]. Therefore, miR-7 is a relevant target to modulate atrophic phenotypes in DM1.

miR-7 biogenesis is regulated by Musashi 2 (MSI2) and Hu R antigen (HuR). MSI2, an RNA-binding protein, binds to the pri-mir-7 terminal loop through the HuR protein inhibiting its processing by Drosha. Consequently, miR-7 biogenesis is inhibited by MSI2/HuR complex [20]. MSI2 was recently reported to be upregulated in DM1 samples, thus leading to excessive miR-7 inhibition [21]. Considering these data, we hypothesized that modulation of MSI2 to regulate miR-7 offered an opportunity to recover atrophic phenotypes in DM1.

Kumar et al. 2017 demonstrated that oleic acid (OA), a natural monounsaturated fatty acid produced by plants and animal cells, binds to MSI2 at its N-terminal RNA recognition motif 1 (RRM1), producing an allosteric conformational change in the protein that prevents it from binding to pri-mir-7-1. Thus, OA treatment in HeLa cells enhanced miR-7 biogenesis [22]. Several studies have demonstrated the importance of OA in human health and diseases [23, 24]. For example, OA modulates several physiological functions and has been suggested as a beneficial therapy for inflammatory, immune, and cardiovascular diseases [25, 26]. In the same way, some published studies have evaluated the effects of OA in muscle [27, 28], reporting fatty acids’ ability to modulate skeletal muscle proliferation and differentiation [29, 30]. Specifically, OA was demonstrated to regulate the differentiation of satellite stem cells and the growth and maturation of myotubes [31]. Additionally, OA promotes the differentiation of L6 myoblasts [32] and C2C12 myoblasts [33, 34].

Here, we report low OA levels in different DM1 cells, which contributes to the miR-7 deficit through its reduced repression on MSI2 and participates in cell differentiation alteration. Furthermore, OA supplement to DM1 myoblast was sufficient to restore different phenotypes related to the MSI2-regulated autophagy. Finally, we propose SCD1 deficiency could contribute to decreased OA levels. Globally, MSI2 inhibition through OA would be a therapeutic strategy to increase endogenous miR-7 and therefore inhibit the processes of muscle atrophy and impaired differentiation. Taken together, we describe a new mechanism contributing to muscle dysfunction in DM1.

## Materials and methods

### Lipid extraction and analytical method for the estimation of OA concentration

1 × 10^6^ cells were seeded in a Petri plate (*n* = 3 to 6). After differentiation and OA treatment, cells were resuspended in 225 µL of methanol (15,518,534, Thermo Fisher Scientific, Waltham, Massachusetts, USA) at 4^o^C. Cells lysis was performed for 20 s vortex three times, followed by 1 min in liquid nitrogen. Samples were kept on ice during thawing and were subjected to sonication (3 × 10 s 0.55 cycle 60% amplitude). Then samples were incubated under agitation for 1 h at 4^o^C with 750 µL of cold methyl-*tert*-butyl ether (MTBE) (143,312, Panreac Applichem ITW Reagents, Barcelona, Spain). After that, 750 µL of a solution of 0.1% C_2_H_7_NO_2_ (10,714,391, Thermo Fisher Scientific, Waltham, Massachusetts, USA) was added to each sample and vortexed, followed by a 10 min incubation at room temperature (RT). Samples were centrifuged at 10,000 x *g* for 5 min at 4^o^C to separate the two phases. The upper phase, containing apolar lipids, was transferred to a new tube and dried in a vacuum centrifuge. The pellet was dissolved in 100 µL of methanol. The volume of the lower phase containing polar proteins was measured, and four volumes of cold methanol were added and incubated for 1 h at -20^o^C and then centrifuged at 13,000 x *g* for 10 min at 4^o^C. The pellet was resuspended in 100 µL of RIPA buffer (10,230,544, Thermo Fisher Scientific, Waltham, Massachusetts, USA) plus protease and phosphatase inhibitor cocktails (4,906,837,001, Roche Applied Science, Indianapolis, IN, USA). Total protein was determined in the Tecan Infinite M200 pro plate reader by using a BCA protein assay and BSA as protein standard (10,741,395, Thermo Fisher Scientific Pierce, Grand Island, NY, USA).

The OA concentration was determined using a liquid chromatography-mass spectrometry (LC/MS) system (ACQUITY TQD, Waters, MA, USA). The chromatographic separation was performed using an ACQUITY UPLC C18 Kinetex column (Phenomenex, particle size 1.7 μm; 2.1 mm X 100 mm). The mobile phase was in isocratic mode MeOH: CHCl_3_: H2O (1:1:0.04). The flow rate used was 0.2 mL/min. The mass spectrometer was equipped with a Z-spray electrospray ionization source, and the samples were analyzed with the following conditions: capillary: 3 KV, cone: 40 V, extractor: 5 V, RF Lens: 0.3 V, source temperature: 120 °C, desolvation temperature: 300 °C, cone gas: 25 L/h, desolvation gas: 650 L/h. MS1 parameters were: LM resolution: 13; HM resolution: 13; ion energy: 1. MS2 parameters were: LM resolution: 13; HM resolution: 13; ion energy: 1; multiplier: 650 V. Spectra were acquired in negative ionization selected reaction monitoring (SRM) mode with an inter-channel delay of Spectra of 0.050 s. OA concentration was normalized to the total protein in each sample.

### Cell lines and culture conditions

Transdifferentiated myoblast (TDM) and immortalized myoblast were obtained from the Institute of Myology, Paris, and were cultured as previously described [35]. Briefly, skin and skeletal muscle biopsies were obtained from a DM1-affected female and a healthy male donor (Table 1). Primary fibroblasts from skin biopsies of DM1 and control donors (Table 1) were immortalized by hTERT induction and transduced to conditionally express MyoD (hereafter referred to as primary transdifferentiated myotubes, pTDM) and cultured as previously described [15]. All experiments were carried out in 7-day differentiated cells except the experiment in which fusion index was determined in control cells at 0, 4, 7 and 10-days of differentiation. For fatty acid supplementation, cells were treated with 0.5 or 2 µM of OA, or 2 µM EA (01008 and E4637, respectively, Sigma Aldrich, San Luis, Missouri, USA), dissolved in 1% DMSO, that also served as vehicle-only control, for 24 h in opti-MEM (31,985,070, Thermo Fisher Scientific, Waltham, Massachusetts, USA). After fatty acid treatment, cells were supplemented with differentiation medium for 7 days. To treat cells with LXR (T 0901317, Tocris Bio-Techne, Bristol, UK) 1 × 10^6^ TDM DM1 cells were seeded in a petri plate and differentiation medium was added after 24 h. At day 4 of differentiation LXR was added at a final concentration of 5 µM or 10 µM, 0.8% de DMSO. during 72 h. At day 7 cells were collected for OA determination.

**Table 1:** Cell lines information

Sample ID	Cell type	Condition	CTG repeats	Age	Sex	Ref.
TDM	Immortalized fibroblast	CNT	-	25	male	[35]
TDM	Immortalized fibroblast	DM1	1300	11	female	[35]
Immortalized myoblast	Immortalized myoblast	CNT	-	25	male	[35]
Immortalized myoblast	Immortalized myoblast	DM1	1300	11	female	[35]
pTDM 819	Primary fibroblast	CNT	-	48	female	[15]
pTDM 988	Primary fibroblast	CNT	-	unknown	female	[15]
pTDM 966	Primary fibroblast	DM1	1000	33	male	[15]
pTDM 973	Primary fibroblast	DM1	333	46	male	[15]

### Toxicity assay

DM1 TDM were seeded in 96-well plates (1 × 10^5^ cells/well). After 24 h cells were treated with LXR as described (concentrations ranging from 0.1 µM to 80 µM). Cell viability was measured after 7 days of differentiation. Briefly, 20 µL of MTS/PMS (CellTiter 96® Aqueous Non-Radioactive Cell Proliferation Assay, Promega, Madison, WI) was added to each well with 80 µL of differentiated medium. Cells were incubated for 4 h at 37 °C with 5% CO2 and absorbance at 490 nm was measured using Infinity Pro M200 plate reader. 4 biological replicates were performed for each condition.

### Immunofluorescent methods

Cells were seeded in 24-well plates (3 × 10^5^ cells/well) and were transdifferentiated for 7 days to carry out immunodetection of DESMIN, LC3B, and MF-20. After 24 h of the corresponding treatment, cells were fixed with 4% paraformaldehyde (PFA) for 15 min at RT. Immunodetection of Desmin was carried out with mouse anti-DESMIN (1:50, ab8470, Abcam, Cambridge, UK), goat biotin-conjugated anti-mouse-IgG (1:200, Sigma-Aldrich, San Luis, Missouri, USA) and Streptavidin Fluorescein (1:200, SA-5001-1, Vector laboratories, Newark, California, USA. For LC3B immunostaining rabbit anti-LC3B (1:3000, 51,520, Abcam, Cambridge, UK) and goat anti-rabbit-IgG (H + L) Alexa Fluor Plus 594 (1:200; Invitrogen, Carlsbad, CA, USA) were used. Immunodetections were performed as previously described [21]. Images were obtained with an LSM800 confocal microscope (Zeiss, Jena, Germany) at 200x magnification.

The fusion index was defined as the proportion of nuclei contained within myotubes (> 2 myonuclei) relative to the overall count of nuclei in each condition. The mean count of nuclei per myotube was ascertained by analyzing over 250 nuclei taken randomly from DESMIN-positive cells (5–7 micrographs). The diameter of myotubes was gauged at 5 points spanning the entire length of the tube. 50 myotubes were scrutinized for each distinctive experimental setup. Quantitative analysis was executed using the ImageJ software (NIH). LC3 puncta quantifications was performed using the Ifdotmeter software [36]. We conducted image analyses on myotubes stained with LC3. Briefly, the quantification of LC3 dots or puncta was executed on 15 − 20 images for each specific condition. The aggregate count of LC3 dots per image was adjusted in proportion to the entire area encompassing all the observed myotubes within each image. Measurement of myotube area was carried out using the ImageJ software. Data were expressed as the number of LC3 dots/µm2.er.

In the case of MF20, the immunostaining was performed as DESMIN but using mouse anti-MHC MF20 (1:20, MYH1E, Developmental Studies Hybridoma Bank, Douglas, USA).

For LysoTracker staining, cells were incubated 30 min at 37 °C with 100 nM of LysoTracker RED-DND99 (12,090,146, Invitrogen, Carlsbad, CA, USA), washed with PBS 1x and fixed with 4% PFA for 15 min. After three washes with PBS 1x, cells were mounted in Vectashield (Vector Laboratories, London, UK) with 2 µg/ml DAPI (4’, 6-diamidino-2-phenylindole). Images were acquired in an LSM800 confocal microscope (Zeiss) at 400x magnification.

### Western blotting

Protein extraction, quantification, and immunodetection were performed as in [16, 21]. Membranes were incubated O/N with the corresponding primary antibody dilutions: mouse anti-β-ACTIN (1:5000, A5441, Sigma-Aldrich, San Luis, Missouri, USA), goat horseradish peroxidase (HRP)-conjugated anti-GAPDH (1:3500, sc-365,062, Santa Cruz, Dallas, Texas), rabbit anti-ATG4A (1:1000, #7613, Cell Signaling, Danvers, Massachusetts, USA), rabbit anti-LC3B (1:3000, 51,520, Abcam, Cambridge, UK), rabbit anti-MSI2 antibody (1:1000, EP1305Y, Abcam, Cambridge, UK), mouse anti-P62 (1:1000, 65,416, Abcam, Cambridge, UK), mouse anti-P21 (1:2000, #2946, Cell Signaling, Danvers, Massachusetts, USA), mouse anti-SCD1 (1:1000, 19,862, Abcam, Cambridge, UK). After three washes with 1x PBS-T, membranes were incubated for 1 h at RT with the corresponding secondary antibody dilutions: goat HRP-conjugated anti-rabbit-IgG (1:3500, A0545, Sigma-Aldrich, San Luis, Missouri, USA) or goat HRP-conjugated anti-mouse-IgG (1:5000, B7264, Sigma-Aldrich, San Luis, Missouri, USA). Images were acquired with an ImageQuant LAS 4000 or AMERSHAM ImageQuant 800 (GE Healthcare) and were quantified using ImageJ software (NIH).

### RNA extraction and real-time quantitative reverse transcription PCR (qRT-PCR)

According to the manufacturer’s instructions, total RNA from 1 × 10^6^ cells was extracted from each experimental replicate using the miRNeasy mini kit (217,004, QIAGEN, Hilden, Germany). For miRNA determinations, 2 µL of 5 ng/µL samples were reverse-transcribed with miRCURY LNA RT kit (339,340, Qiagen, Hilden, Germany). miR-7 expression was quantified using specific miRCURY LNA miRNA PCR primers (339,340, Qiagen, Hilden, Germany) and was normalized to *U1* or *U6* small nuclear RNA (snRNA) (339,306, Qiagen, Hilden, Germany) according to the manufacturer’s recommendations. For analyses of relative expression of genes, one microgram of total RNA was digested with DNase I and reverse-transcribed with SuperScript II (18,068,015 and 18,064,014, respectively, Invitrogen, Carlsbad, CA, USA) using random hexanucleotides (11,277,081,001, Sigma-Aldrich, San Luis, Missouri, USA). qRT-PCR was performed using 2 ng of cDNA template with 5x HOT FIREPol EvaGreen qPCR mix plus (ROX) (08-24-000 S, Solis BioDyne, Tartu, Estonia) and specific primers. Gene expression was normalized to *GAPDH* and *GPI*. The primers used were: *GAPDH* (fwd CATCTTCCAGGAGCGAGATC; rev GTTCACACCCATGACGAACAT), *GPI* (fwd CAGGGCATCATCTGGGACAT; rev TCTTAGCCAGCTGCTTTCCC) and *MSI2* (fwd GCAGACCTCACCAGATAGCCTT; rev AAGCCTCTGGAGCGTTTCGTAG); *HMGR* (fwd TGCTTG CCGAGCCTAATGAAAG; rev AGAGCGTTCGTGGGTCCATC); *FAS* (fwd AAGGCTTTCGTGGGTCCATC; rev GATGCCAATTACGAAGCAGTTG); *PGC1-α* (fwd CCAAAGGATGCGCTCTCGTTCA; rev CGGTGTCTGTAGTGGCTTGACT); *RNF145* (fwd GCAGGTTGTTCATCGGGCATTC; rev GGCTTACAGCACGGAAGTGTTTC); *ABCA1* (fwd CAGGCTACTACCTGACCTTGGT; rev CTGCTCTGAGAAACACTGTCCTC); and *SCD1* (fwd TGTGGTGAAGTTGATGTGCCAGC; fwd CCTGGTTTCACTTGGAGCTGTG).

qRT-PCRs were performed in a Step One Plus PCR system (Applied Biosystems, Foster City, CA, USA). Three experimental replicates and three repeats per biological sample were performed. Expression levels were normalized to the mean of reference genes using the comparative cycle threshold (Ct) method (2^−ΔΔCt^) [37].

### Statistical analyses

Graphical outputs from statistical analyses used GraphPad Prism 9 software. In all experiments, we assumed that all parameters followed a normal distribution to compare normalized data means. For comparisons of two conditions, a two-tailed Student’s t-test (α = 0.05) was applied with Welch’s correction when necessary. For comparisons of more conditions, one-way ANOVA (α = 0.05) was applied, and Tukey’s HSD post hoc test when necessary. The correlation was measured with Pearson’s correlation coefficient.

## Results

### A marked reduction of OA levels is detected in DM1 in vitro

We previously proposed that MSI2 overexpression contributed to wasting phenotypes through repressing miR-7 biogenesis that hyperactivated muscle autophagy [21]. Considering the previously described impact of OA in MSI2 function during miR-7 maturation [22], we tested the hypothesis that OA deficits could impinge on miR-7 levels in DM1 by quantifying OA by mass spectrometry in different DM1 muscle cells.

**Fig. 1:**
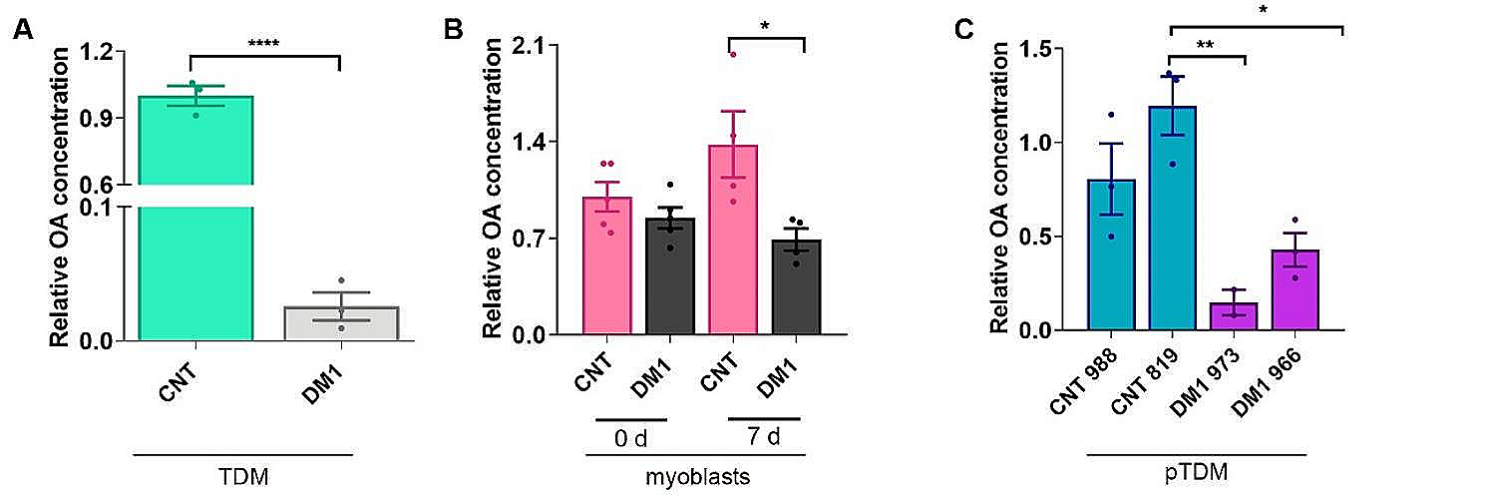
OA levels are decreased in DM1 cells. OA levels were quantified by LC/MS in (**A**) healthy controls (CNT) and DM1 TDM, (**B**) undifferentiated (0 d) and differentiated (7 d) myoblasts obtained from a DM1 patient and unaffected control, and (**C**) two DM1 (973 and 966) and two control pTDM (988 and 819) transdifferentiated for 7-day into myotubes. In A) and C), OA values are shown relative to levels in control muscle cells, and in b) are relative to CNT myotubes prior to differentiation. The bar graphs show mean ± S.E.M. *P < 0.05, **P < 0.01, **** *P* < 0.0001 according to Student’s t-test. In all cases, experimental replicates are *n* = 3–4

Firstly, we measured OA in DM1 fibroblasts expressing 1300 CTG repeats [40] and transdifferentiated by doxycyclin-induced MyocddsD expression into multinucleated myotubes (hereafter referred as transdifferentiated myotubes, TDM) for 7 days (Fig. 1A). The results showed a 40-fold reduction in OA in DM1 TDMs compared to healthy controls. The same determination was performed on DM1 and control immortalized myoblasts at 0 and 7 days of differentiation. In this case, OA levels were halved compared to controls after 7 days of differentiation with no preexisting differences at day 0 (Fig. 1B). Finally, OA levels were analysed in two primary fibroblast cell lines from DM1 patients and two unaffected donors, transdifferentiated for 7 days into myotubes (hereafter referred as primary transdifferentiated myotubes; pTDM; Fig. 1C). In this case, more than 6 and 2-fold reduction was observed in line 973 and 966, respectively, compared to controls. Thus, we confirmed a significant reduction in OA levels in four cell lines derived from 3 DM1 patients (note TDM and immortalized myoblasts from the same donor; Table 1) compared to their corresponding controls.

### OA derepresses miR-7 biogenesis with stable MSI2 levels

**Fig. 2:**
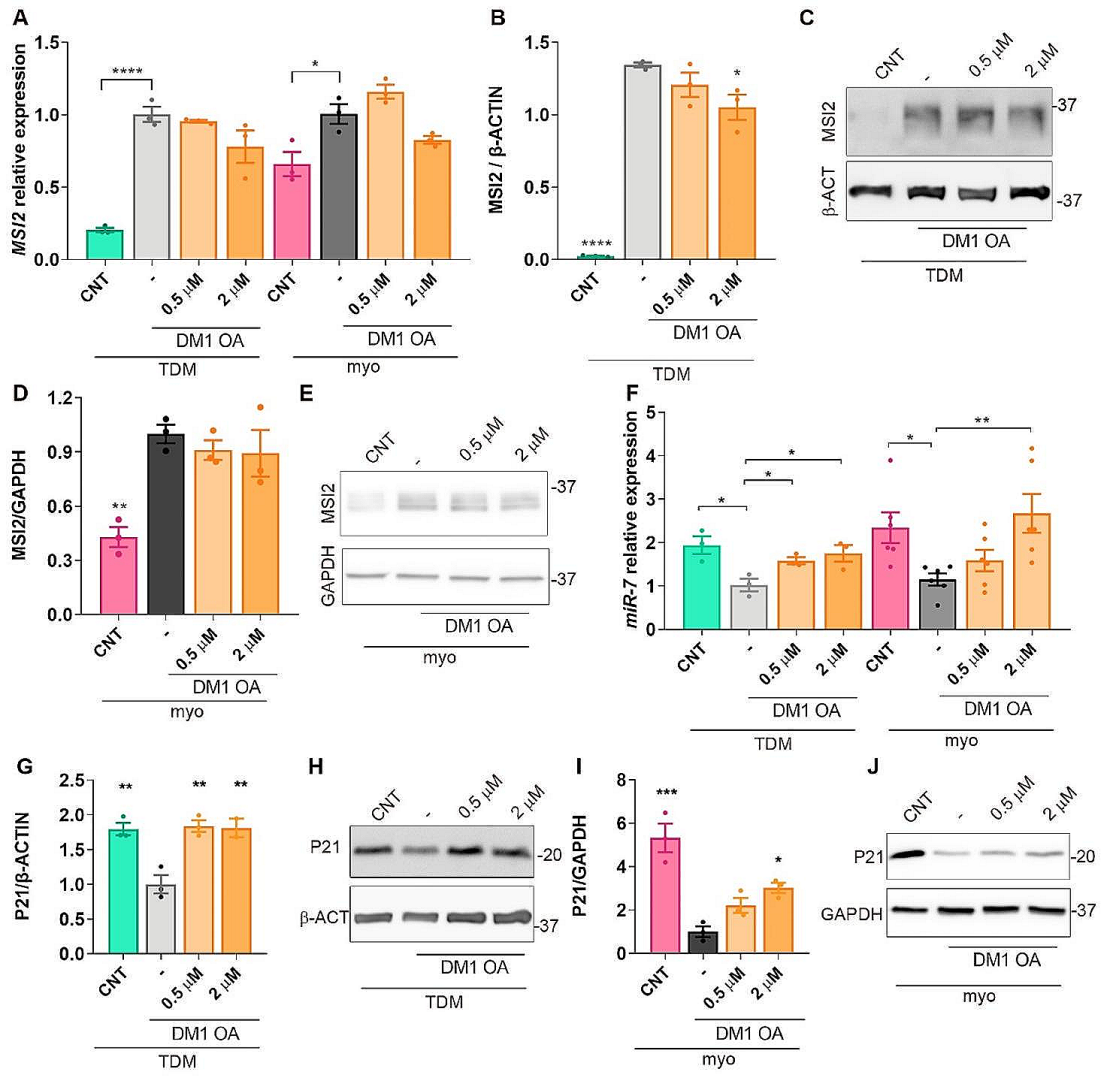
Inhibition of MSI2 activity by OA increases levels of MSI2 targets. Quantification of *MSI2* transcripts (**A**) or protein (**B**-**E**) in 7-day-differentiated TDM (**B**, **C**) and immortalized myoblasts (**D**, **E**) treated with 1% DMSO as vehicle (-) or OA at the indicated concentrations (*n* = 3). Representative western blots are also shown (**C**, **E**). (**F**) The same samples as in (**A**) were used to determine relative miR-7 levels by RT-qPCR. (**G**-**J**) Quantification of P21 relative levels by western blot in the indicated conditions. *MSI2* transcripts were normalized to endogenous expression of the mean of *GAPDH* and *GPI.* miR-7 quantification is relative to endogenous *U1* and *U6* levels. Asterisks indicate statistically significant differences between DM1 cells treated with DMSO (-) and the conditions indicated in the graphs. The bar graphs show mean ± S.E.M. *P < 0.05, **P < 0.01, **** *P* < 0.0001 according to one-way ANOVA test

The binding of OA to the N-terminus of MSI2 causes an allosteric change that prevents MSI2 binding to the HuR-MSI2-pri-miR-7-1 complex, thereby derepressing miR-7 biogenesis [22]. Having confirmed the abnormally low levels of OA in DM1 cells, we hypothesized that OA supplementation would enhance miR-7 levels by partially interfering with MSI2-mediated repression. TDM and immortalized myoblasts were grown in a medium supplemented with 0.5 or 2 µM OA. To confirm the efficacy of the treatment, OA was determined in cells after exposure to the highest dose of the fatty acid. For TDMs, the increase in OA levels in cells at 0 and 7 days of differentiation was 1.57 and 1.50 µM, respectively. This variation was slightly higher for immortalized myoblasts treated in parallel, reaching 2.08 and 2.02 µM, thus, confirming the effectiveness of the treatment. Exposure of DM1 cells to 0.5 or 2 µM OA demonstrated that MSI2 transcript and protein levels were not significantly affected by treatment (Fig. 2A-E; Supplementary Fig. 1). However, we observed a significant increase in miR-7 expression in TDMs at both concentrations tested, and at the highest concentration in the case of immortalized myoblasts (Fig. 2F), consistent with the OA´s allosteric role upon binding MSI2. In all cases, miR-7 expression upon OA treatment reached values similar to those detected in CNT cells. These results reinforce the deleterious effect of increased MSI2 in DM1 where, in addition to being upregulated [21], its inhibitory activity on miR-7 maturation from pri-miR-7-1 synergizes with the abnormally low levels of OA we detect in DM1 cell lines.

It was previously described that MSI2 overexpression was sufficient to reduce P21 levels and that its inhibition had the opposite effect over P21 [38, 39]. Therefore, to confirm that inhibiting MSI2 regulatory activity by OA affected its targets, we quantified the levels of P21 in TDM and immortalized myotubes exposed to an OA-enriched medium (Fig. 2G-J, Supplementary Fig. 2). The results showed that P21 was significantly reduced in DM1 cells, as expected under conditions where MSI2 is hyperactivated. Upon OA treatment of DM1 TDMs, P21 was derepressed, reaching levels similar to controls. Furthermore, in the case of immortalized myotubes, where P21 levels in DM1 cells were around 5-fold lower than in controls, a significant two- to three-fold increase over untreated DM1 was observed after OA treatment.

### Excessive muscle autophagy in DM1 is restored upon OA treatment

**Fig. 3:**
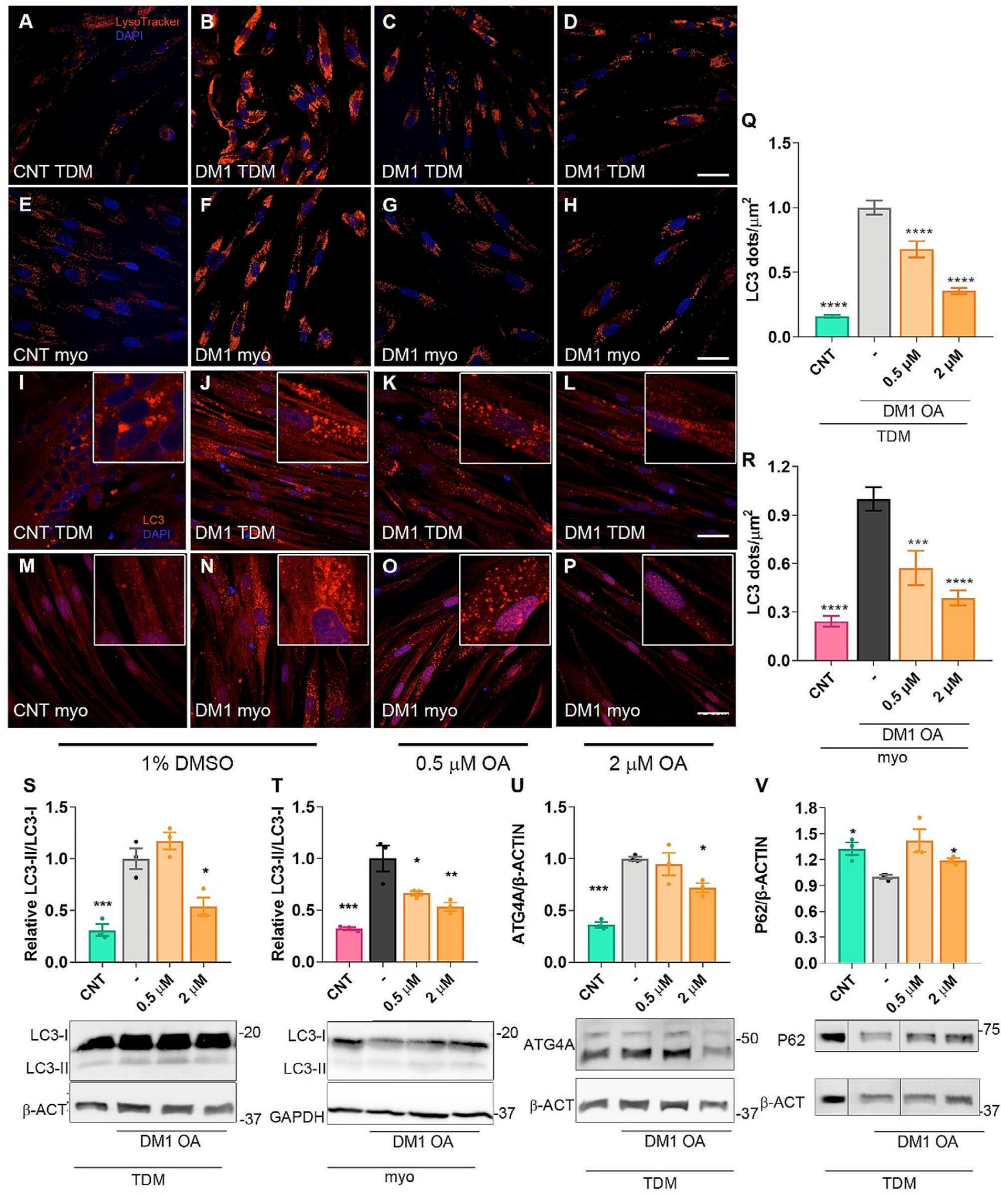
OA represses autophagy in DM1. Representative Confocal images of LysoTracker staining (red, A-H) and LC3 immunodetection (red, I-P) in TDM (A-D, I-L) and immortalized myoblasts (E-H, M-P) treated for 24 h with 1% DMSO or OA at a final concentration of 0.5 or 2 µM and differentiated for 7 days. (Q, R) Quantification of LC3 puncta per square micrometer (*n* = 20 to 25 images per condition) in TDM (Q) and immortalized myoblasts (R). (S-V) Quantification and representative western blots of immunoreactive bands for LC3 in control and DM1 TDM (S) or immortalized myoblasts (T). (U, V) Representative western blots, with indication of molecular weight sizes to the right in kDa and quantification of total ATG4 and P62 in protein extracts from healthy control (CNT), DM1 TDM untreated or treated with the indicated concentrations and differentiated for 7 days. DM1 cells were treated with 1% DMSO as a control (-) or 0.5 or 2 µM OA for 24 h (*n* = 3). Each condition was compared to DM1 cells treated with 1% DMSO. The scale bar equals 20 μm. Nuclei were counterstained with DAPI. The bar graphs show mean ± S.E.M. *P < 0.05, **P < 0.01, ∗∗∗*P* < 0.001, and ∗∗∗∗*P* < 0.0001 according to one-way ANOVA test

It was previously demonstrated that abnormally low levels of miR-7 in DM1 muscle cells contribute to the pathological increase in autophagy and that its modulation was sufficient to impact this catabolic pathway [16]. Therefore, after confirming the de-repression of miR-7 biogenesis upon OA treatment, we investigated autophagy status in OA-treated TDM and immortalized myoblasts by staining the cells with LysoTracker. This acidotropic dye marks the acidic cellular compartments, namely, lysosomes and autophagolysosomes. Compared to control counterparts, we confirmed stronger signal indicative of autophagy overactivation in DM1 cells. Notably, a substantial signal reduction was detected in cells that underwent OA treatment at either 0.5 or 2 µM (Fig. 3A-H), despite still being higher than that in control cells. Additionally, LC3-I and LC3 conjugated to membrane-bound phosphatidylethanolamine (LC3-II) were detected by immunofluorescence. DM1 cells (TDM and immortalized myoblasts) showed a strong signal with a punctate pattern corresponding to LC3-II in autophagosomes [40] (Fig. 3I-P). In healthy controls, the signal was less intense, more diffuse, and predominantly cytoplasmatic, indicating the presence of soluble LC3-I, and reduced autophagic levels. In OA-treated DM1 cells, the signal lost intensity, and the presence of dots dropped notably. We quantified LC3-II dots per unit of area in each experimental condition (Fig. 3Q, R). Results revealed a significant 5 and 4-fold increase in the presence of LC3-II in DM1 TDM and immortalized myoblasts, respectively, compared to their corresponding controls that significantly dropped in OA-treated cells, reaching levels similar to those in control cells at the highest OA tested dose.

Complementary to the immunofluorescence assessment of autophagosome-associated and soluble LC3 (LC3-II/LC3-I ratio), we quantified their levels by western blot. Results confirmed the overactivation of the pathway in DM1 TDM as well as in DM1 myoblasts, and the LC3-II/LC3-I ratio was restored to close to normal when cells were cultured at the highest OA dose (Fig. 3S, T, Supplementary Fig. 3). Importantly, this reduction was in a dose-dependent manner in the case of TDM where no effect was detected in cells treated with 0.5 µM OA, but a substantial drop in the ratio was observed upon OA treatment at the highest dose. When DM1 myoblasts were supplemented, the parameter was reduced to almost half the values in untreated cells, with a slight difference between both concentrations of OA tested. To confirm the effect of OA supplement on this pathway, we additionally evaluated ATG4A, which besides being directly involved in the autophagic pathway, is translationally repressed by miR-7 [19]. Results showed that ATG4 was overexpressed (3-fold) in DM1 TDM, and protein levels significantly decreased upon OA treatment at the highest dose (Fig. 3U; Supplementary Fig. 4A). Finally, we evaluated the impact of OA treatment on the P62 scaffold protein (Fig. 3V; Supplementary Fig. 4B). Low levels of autophagic activity lead to the accumulation of P62, as autophagy itself degrades the protein [41, 42]. Our results confirm the significant reduction of this protein in DM1 TDM cells and its increase toward control-like amounts upon supplementing OA in the cells medium.

### OA supplementation enhances DM1 muscle cell differentiation

**Fig. 4:**
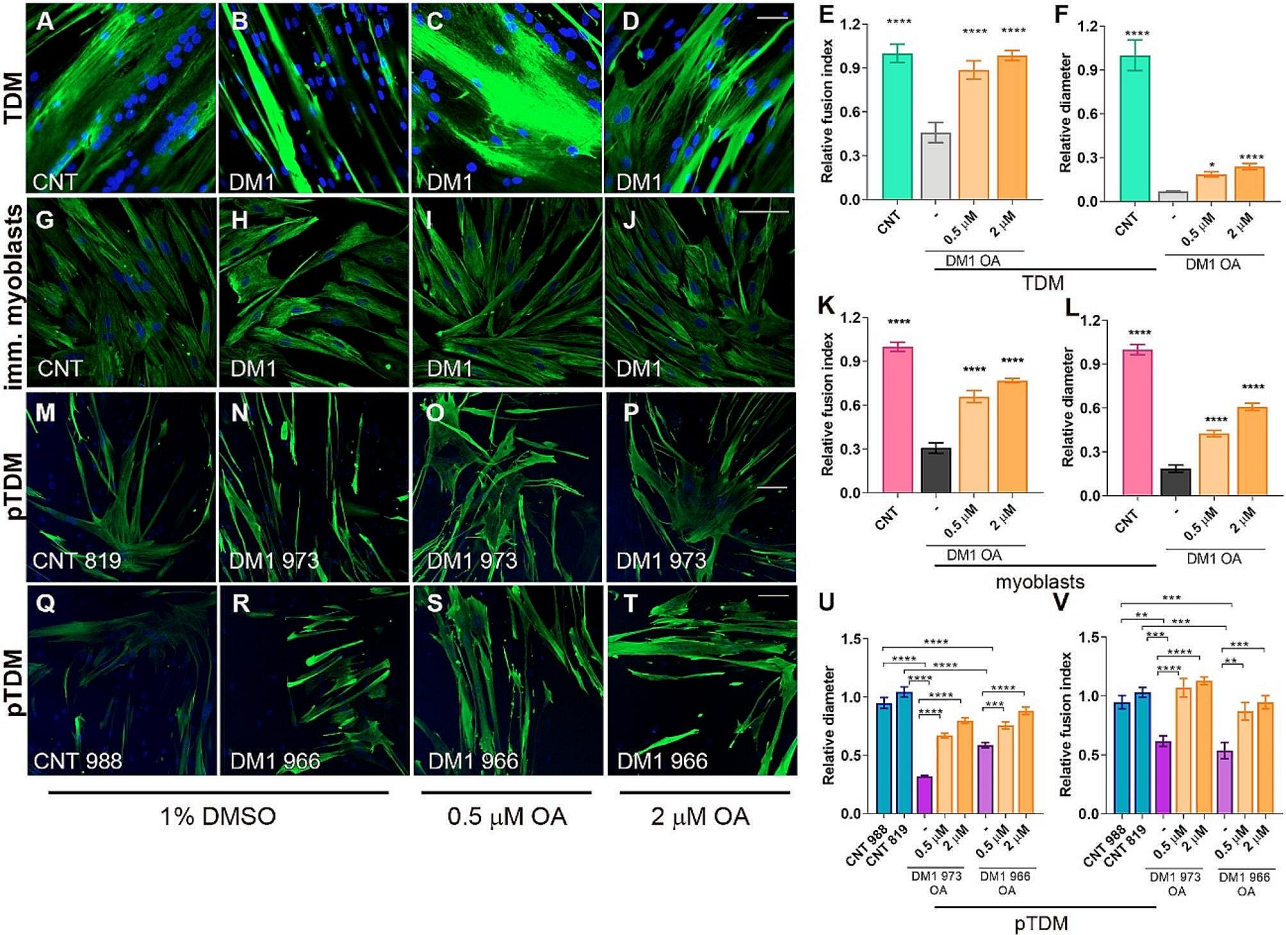
OA treatment promotes muscle cell differentiation parameters in DM1 cells. Representative confocal microscopy images of immunofluorescence using an anti-Desmin antibody (green) in CNT and DM1 TDM (**A**-**D**), immortalized myoblasts (**G**-**J**), and the indicated control (**M** and **Q**) and DM1 pTDMs (N-P and R-T). The DM1 lines were treated with 1% DMSO, 0.5, or 2 µM of OA for 24 h. CNT cells were treated with 1% DMSO. In all cases, cells were differentiated for 7 days. Nuclei were counterstained with DAPI. The scale bar equals 100 μm. Fusion index (E, K,U) and myotube diameter (F, L,V) were obtained for each cell line (*n* = 10 to 15 images per condition). All comparisons were made against DM1 cells treated with the vehicle (- in the graphs). The bar graphs show mean ± S.E.M. *P < 0.05, ***P < 0.001, **** *P* < 0.0001 according to one-way ANOVA test

Given the background linking OA to cell proliferation and differentiation [28, 29, 34], we proposed that its low levels could be one of the reasons why DM1 model cells have differentiation defects, as previously reported [3, 16, 35]. To test this hypothesis, the different cell lines (Table 1) were exposed to 0.5 or 2 µM of OA and stained for Desmin, a marker of myotubes. Then, the fusion index and diameter of 7-day-differentiated myotubes were determined. In immortalized healthy myoblasts and TDMs, we determined the fusion index and myotube diameter in parallel at differentiation times of 0, 4, 7, and 10 days. Results demonstrated a similar evolution of the fusion index in both lines with an exponential increase until 7 days of differentiation, where values close to 100% were reached and remained stable until day 10. Regarding the myotube diameter we observed an increase in the values of this parameter as days of differentiation increased while for TDM at day 10, a marked drop (*p* > 0.0001) in this parameter was observed compared to the previous time point, 7d. (Supplementary Fig. 5A-J). Overall, these results demonstrate that under our experimental conditions, at 7 days of differentiation, both myoblasts and TDMs behave similarly in terms of their differentiation profile. In all cases, the treatment was found to improve the phenotypes significantly. Specifically, for TDM (Fig. 4A-F), DM1 cells showed their fusion capacity reduced to less than half of that observed in control cells, which upon OA treatment, reached values similar to those in the control condition (Fig. 4E). As for myotube diameter, also reduced in DM1 TDM, this parameter improved by 2 to 4 times upon OA supplementation compared to untreated DM1 TDM (Fig. 4F). However, the size of the cells was still significantly smaller than control TDM, and the rescue was far from complete. Results were reproduced when the experiment was carried out in DM1 immortalized myoblasts (Fig. 4G-L) and DM1 pTDMs (Fig. 4M-V). In pTDM, although both fusion index and diameter phenotypes were milder than in the other cell lines, they still significantly ameliorated upon OA treatment. We also performed MF20 immunofluorescence in TDMs as a marker of late differentiation of myotubes [43]. The analysis of the images revealed a pronounced defect in the terminal differentiation of DM1 cells, as no stained cells with anti-MF20 antibody were detected. However, following treatment with OA, this value increased in a dose-dependent manner, reaching 60% in cells treated with the highest concentration of OA, with values even exceeding those obtained for control cells (Supplementary Fig. 5J-N). Similar to fusion index and diameter, OA treatment was sufficient to improve this phenotype significantly.

### DM1 muscle cell differentiation rescue by OA is specific

**Fig. 5:**
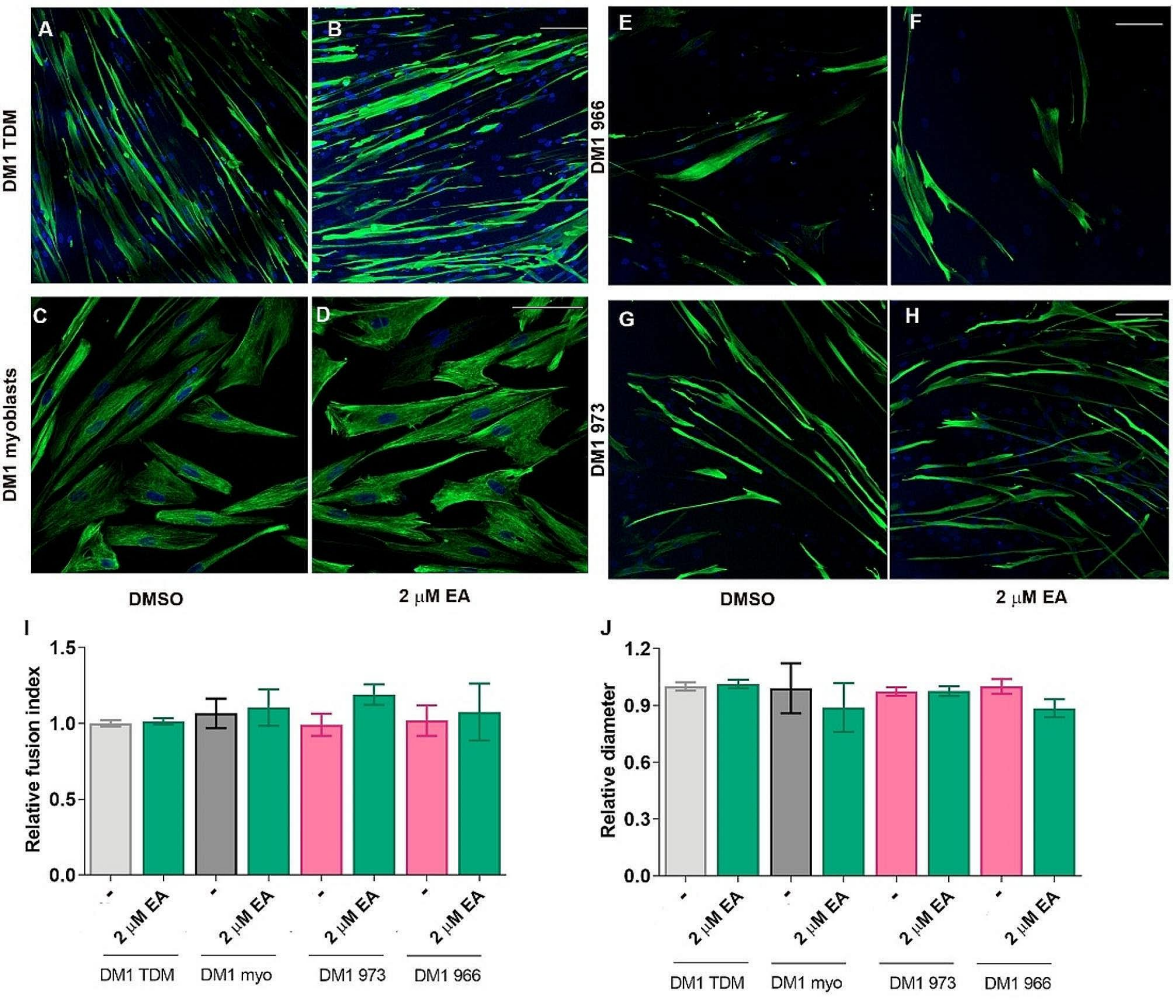
The OA isoschizomer EA fails to rescue DM1 muscle cell differentiation defects. Representative immunofluorescence stainings of Desmin (green) in 7-day-differentiated DM1 TDMs (**A**, **B**), DM1 immortalized myoblasts (**C**, **D**), and two lines of primary fibroblast transdifferentiated into myotubes (**E**, **F**, and **G**, **H**) treated with 1% DMSO (**A**, **C**, **E**, **G**) or 2 µM EA (**B**, **D**,**F**, **H**). Two parameters were analyzed for each condition, the fusion index (I) and the myotube diameter (**J**), shown relative to the DMSO condition. Nuclei were stained in blue (DAPI). The scale bar equals 100 μm. The bar graphs show mean ± S.E.M. No significant differences were detected according to Student’s t-test

To ascertain whether the phenotype reversal is specific to OA supplementation and not attributable to a general deficit of fatty acids, we assessed the impact of supplementing the medium with elaidic acid (EA). The EA fatty acid is a *trans*-isoform of OA with the same molecular weight, similar refractive index, and molar aqueous solubility, but its ω-9 double bond is *trans* rather than *cis* [44], and cannot interfere with MSI2 activity [45]. In fact, it has been demonstrated that OA binds directly to MSI2 with an inhibition constant of 1.2 µM. However, this parameter could not be obtained for EA as its interaction with MSI2 is minimal when tested at similar concentrations to those used with OA [45, 46]. Additionally, recent results have elucidated the tridimensional structure of the interaction between MSI2 and OA [47]. Thus, EA is an appropriate control to demonstrate the specificity of the OA treatment. For that purpose, we treated DM1 lines with 2 µM EA, the highest concentration at which the OA effect was studied. We immunodetected DESMIN with a specific antibody in the 4 available DM1 patient-derived lines in which we demonstrated that OA supplementation was sufficient to improve both fusion capacity and cell size significantly. Notably, no significant differences were detected in either parameter between cells treated with DMSO and 2 µM of EA (Fig. 5A-J).

**Fig. 6:**
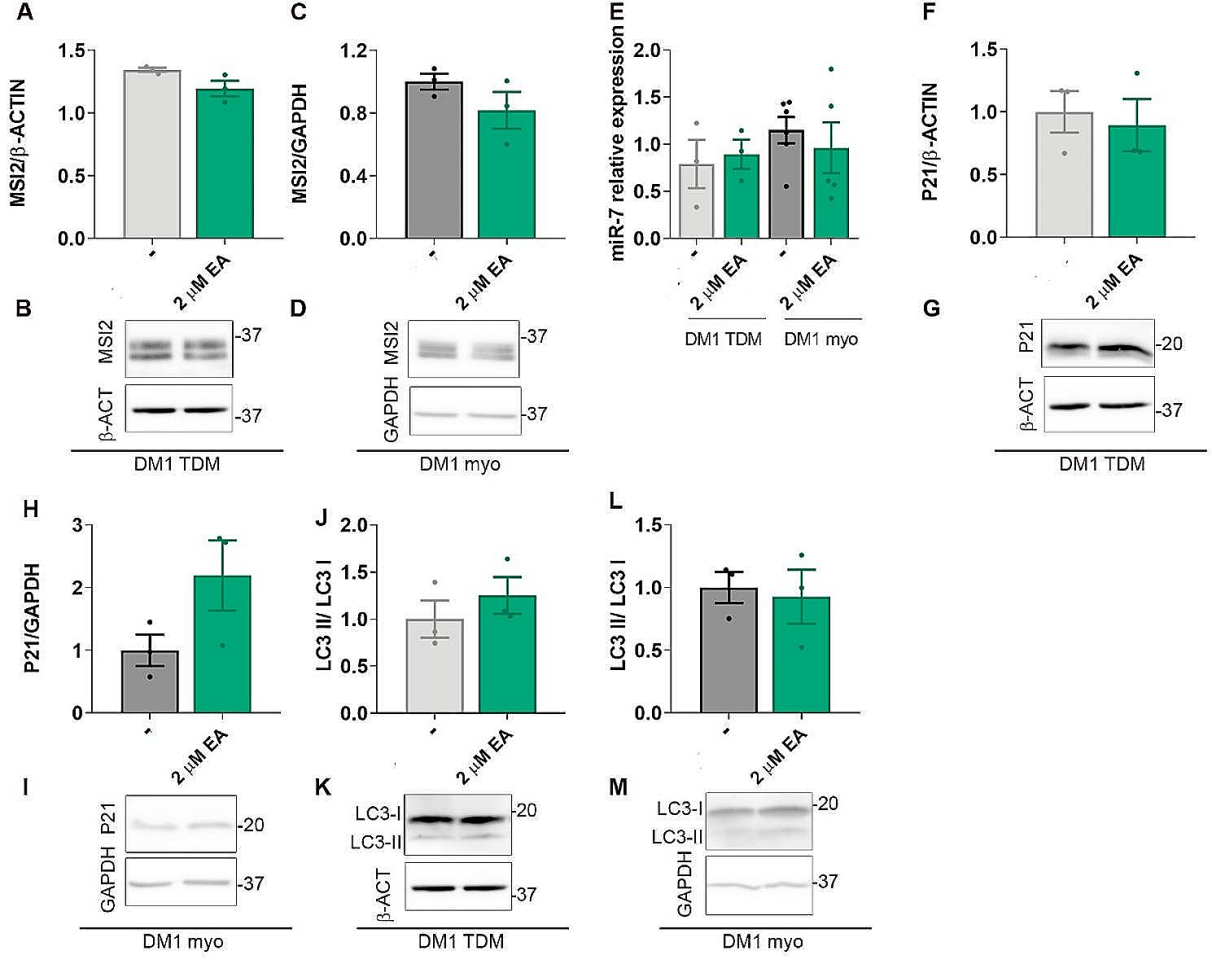
EA fails to inhibit several MSI2-related molecular functions. (**A**-**D**) Quantification and representative blots of immunoreactive bands for MSI2 in protein extracts from DM1 TDMs (**B**, **C**) or immortalized myoblasts (**D**, **E**) supplemented for 24 h with 1% DMSO or 2 µM EA. (**E**) Quantification of the relative expression of miR-7 relative to endogenous U1 and U6 levels (*n* = 3–5) in DM1 TDMs and immortalized myoblasts treated for 24 h with 1% DMSO or 2 µM EA and differentiated for 7 days. *MSI2* transcripts were normalized to endogenous expression of the mean of *GAPDH* and *GPI* (*n* = 3). (F-M) Quantification and representative blots of P21 levels and LC3-II/LC3-I ratio in DM1 TDMs (**F**, **G**,**J**, **K**) or immortalized myoblasts (H, I,L, M) in the same experimental conditions as in (**A**-**D**) (*n* = 3). The bar graphs show mean ± S.E.M. No significant differences were detected according to Student’s t-test between DMSO and EA-treated cells

Effects of EA were characterized at the molecular level in DM1 TDMs and immortalized myoblasts differentiated for 7 days. First, treatment with the OA isoschizomer EA did not affect MSI2 protein levels (Fig. 6A-D, Supplementary Fig. 1). Nevertheless, as was the case after OA treatment, there was a possibility that EA was hindering the interaction of MSI2 with its targets. However, we confirmed that both miR-7 and P21 levels remained unchanged after exposure of both cell lines to EA supplemented medium (Fig. 6E-I, Supplementary Fig. 2). Consistent with these results, we also detected no effect on the autophagy marker LC3-II/LC3-I ratio, which was sensitive to OA treatment of DM1 muscle cells (Fig. 6J-M, Supplementary Fig. 3). Our results confirm that MSI2 inhibition is OA-specific, which is in line with results obtained by independent groups that showed that the affinity of EA for MSI2 was much lower than that of its isoschizomer, OA [45].

Additionally, we evaluated the expression of *peroxisome proliferator-activator receptor γ coactivator 1-α* (*PGC1α*) whose expression depends on OA levels in skeletal muscle [48] and those of *fatty acid synthase* (*FAS*, also known as *FASN*) and *3-hydroxy-2-methylglutaryl-CoA reductase* (*HMGR*) whose upregulation occurs after EA supplementation in HuH-7 hepatic cells [49]. However, the data showed that the expression of these genes remained stable after supplementation of the medium with either OA or EA (Supplementary Fig. 7). These results suggest that the OA effect at the concentrations tested in DM1 cells may be due specifically to its interaction with MSI2 and not to other canonical pathways related to lipogenesis and fatty acid oxidation where similar in vitro experiments use concentrations up to 250-fold higher than those used in this work [50].

### SCD1 expression is strongly repressed in DM1 muscle cells

**Fig. 7:**
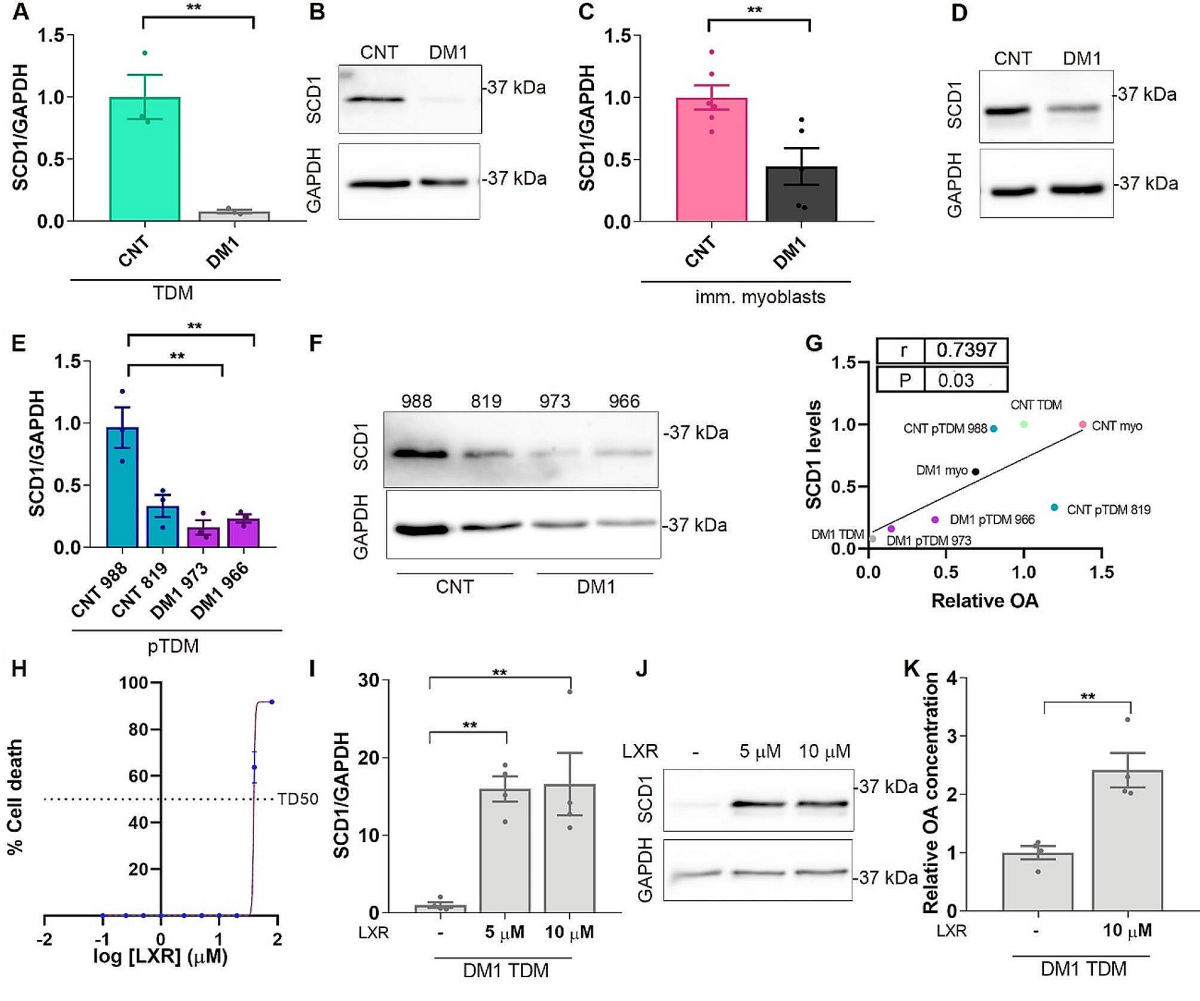
Expression of the critical OA synthesis enzyme SCD1 is reduced in DM1 cells. (**A**-**F**) Quantification and representative immunoblots of SCD1 in control and DM1 TDMs (**A**, **B**) immortalized myoblasts (C, D), and in two different lines of control (988 and 819) and DM1 (973 and 966) pTDMs. (**G**) Pearson’s correlations between the relative SCD1 protein levels and OA levels in control and DM1 TDM (CNT/DM1 TDM), immortalized myoblasts (CNT/DM1 myo) or primary transdifferentiated myoblasts (CNT/DM1 pTDM). (H) Cell growth inhibition assay by 3-(4,5-dimethylthiazol-2-yl)-5-(3-carboxymethoxy-phenyl)-2-(4-sulfophenyl)-2 H-tetrazolium, inner salt (MTS) method. 7-day-differentiated DM1 TDM were treated with a range of concentrations of LXR (*n* = 4). TD50 (39.03 µM) was obtained using the least-squares non-linear regression model. (**I**, **J**) Determination of relative SCD1 levels in DM1 TDM supplemented with 5 or 10 µM LXR. (**K**) Quantification of OA levels by LC/MS in LXR-treated DM1 TDM. In all cases, cells were differentiated for 7 days. Protein was normalized to endogenous GAPDH. The bar graphs show mean ± S.E.M. **P < 0.01 according to Student’s t-test. In all cases, 3 < *n* < 6

Having confirmed the deficit of OA in DM1 muscle cells and the therapeutic potential of its supplementation, we investigated the causes that could be contributing to its deficit. Unlike other fatty acids that come exclusively from the diet, OA can be synthesized *de novo* by stearoyl-CoA desaturase 1 (SCD1) from stearic acid [51]. To assess the hypothesis that endogenous production of OA is impaired in DM1 cells, we quantified this enzyme’s levels in the cell lines in which we had previously confirmed OA deficit. The results demonstrated a decrease of more than 14-fold in DM1 TDMs compared to controls (Fig. 7A, B, Supplementary Fig. 7); this reduction was also significant in immortalized myoblasts although milder than in the previous case (2-fold reduction) (Fig. 7C, D, Supplementary Fig. 7). Finally, in the case of pTDM, the enzyme was strongly reduced (between four and six times) in patient-derived pTDM when compared to lines 988. However, no significant differences were detected when comparing cells derived from patients to those from controls 819 (Fig. 7E, F, Supplementary Fig. 7). Consistently, the relative expression of SCD1 protein and OA levels correlated positively and significantly in all the DM1 muscular cell lines (*r* = 0.74, *p* = 0.03), (Fig. 7G). To confirm that the OA deficit in DM1 conditions was at least partially due to SCD1 deficiency, we proceeded to supplement the medium of the cells with a liver X receptor (LXR) agonist. LXR is a nuclear receptor and transcription factor that directly and positively regulates SCD1 [52, 53]. First, we evaluated the toxicity of this compound in DM1 TDM differentiated for 7 days to establish a safe range of working concentrations (Fig. 7H). It was determined that the TD50 was 39.03 µM. Thus, we proceeded to supplement the cells with 5 or 10 µM LXR. After treatment, SCD1 levels were determined and its activation was confirmed by detecting a more than 15-fold increase regardless of the LXR dose. Similar results were observed both at protein and mRNA levels (Fig. 7I, J, Supplementary Fig. 8). SCD1 activation correlated with a more than 2-fold increase in OA levels in DM1 TDM (Fig. 7K, Supplementary Fig. 7). As controls, we quantified transcripts of *ATP binding cassette subfamily A member 1* (*ABCA1*), *ring finger protein 145* (*RNF145*), and *FAS*, also regulated by LXR transcription factor and involved in the lipids metabolism (Supplementary Fig. 8) [54]. At the tested doses, LXR agonist was sufficient to induce *ABCA1* and *RNF145* expression. However, no effect on *FAS* transcripts was detected, supporting the idea that response to LXR-agonist treatment was dose-dependent and did not have broad effects at the tested concentrations.

Together, these results demonstrate that a deficit in SCD1 enzyme levels contributes to low levels of OA present in DM1 muscle cells.

## Discussion

We have previously demonstrated that miR-7 is downregulated in DM1 and that MSI2 overexpression contributes to this reduction [16, 21]. OA inhibits the RNA-binding ability of the MSI1 and MSI2 proteins by interacting with their N-terminal RNA recognition motif 1(RRM1) and inducing an allosteric change to the MSI conformation, thus preventing binding to target RNAs, including miR-7 biogenesis precursors [45]. Indeed, HeLa cell treatment with OA was sufficient to induce mature miR-7 production due to the inhibitory role of OA on MSI2 activity [22]. Determination of OA levels in DM1 myotubes demonstrated a marked deficit of OA levels. Therefore, two seemingly independent contributions exist to reducing miR-7 levels and enhancing its pathological effects. On the one hand, the over-expression of MSI2 in DM1 and, on the other hand, the deficit of OA potentiates the inhibitory activity of MSI2 on miR-7. Altered OA levels have been linked to pathologies of many different etiologies, such as Alzheimer’s disease [55], cardiovascular disease [56], or cancer [57]. There are more than 150 clinical trials for various pathologies in which OA is being assessed as therapy [58]. Thus, we report the reduced OA levels and its therapeutic potential in myotonic dystrophy.

**Fig. 8:**
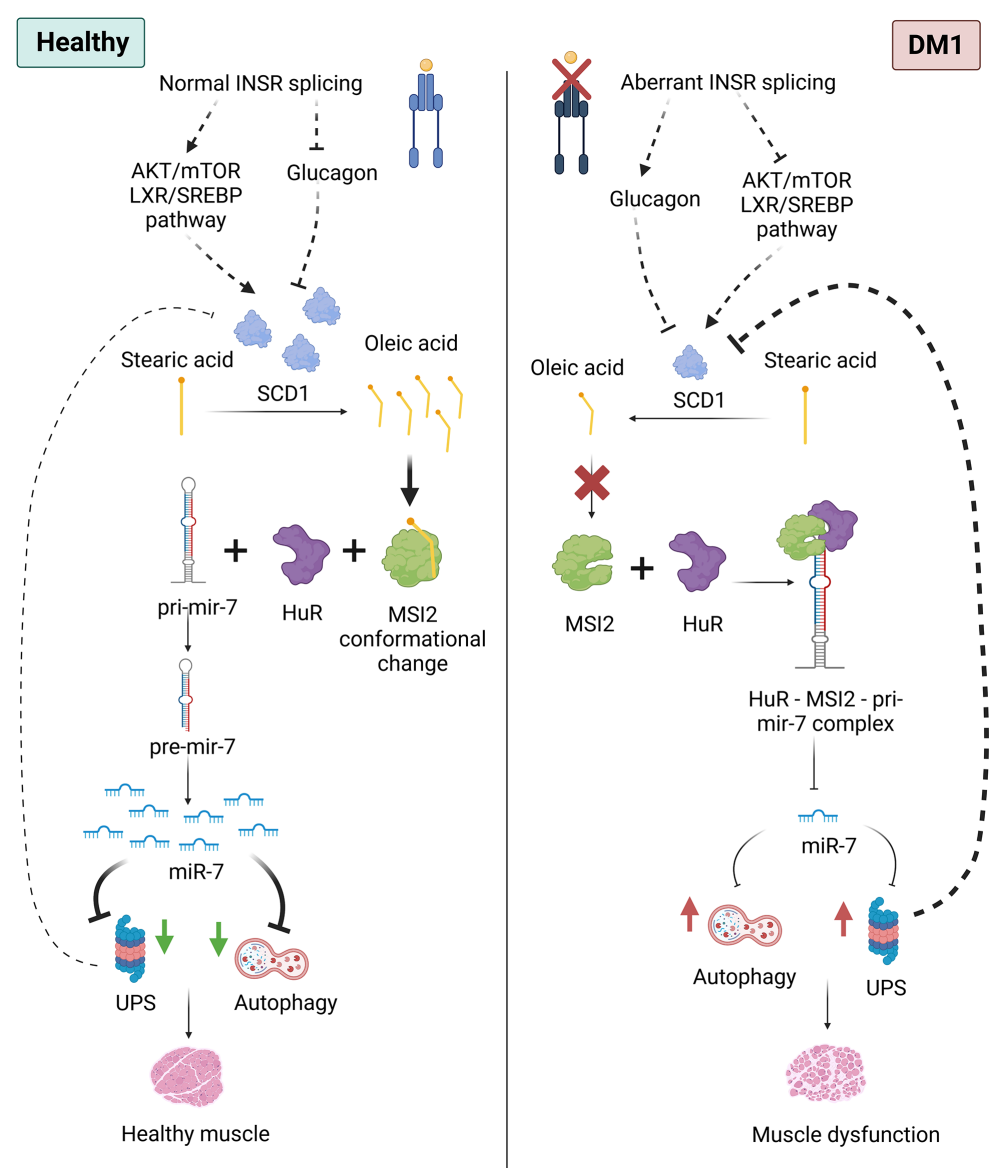
Schematic representation of the involvement of OA in DM1. Reduced levels of SCD1 in DM1, possibly promoted by high UPS activity and/or aberrant *INSR* splicing, lead to a deficit of OA. Low OA levels allow the binding of the MSI2-HuR complex to pri-mir-7, thereby inhibiting the generation of the mature miR-7, which derepresses autophagy and UPS leading to the characteristic imbalance of muscle homeostasis of DM1 by potentiating these catabolic pathways. On the other hand, high OA levels release HuR-MSI2 complexes from the pri-mir-7 precursors, allowing normal levels of the miRNA and repression on autophagy and UPS. Figure created with BioRender.com

Our results suggest a defect in OA biosynthesis as the leading cause of its low levels. OA reaches cells supplied from the diet or is endogenously produced from the desaturation of stearic acid by the SCD1 enzyme that converts saturated fatty acids into their monounsaturated counterparts by introducing a double bond between positions 9 and 10 of the carbon chain [59]. Independent studies observed that SCD1 levels increase during myogenesis when satellite cells differentiate into myotubes [60], reducing stearic acid levels while OA levels increase. These data are consistent with ours, where the defective muscle differentiation observed in DM1 myoblasts [16] could be related to the abnormally low levels of SCD1 and reduced OA. Indeed, OA levels in myoblasts at 0 days of differentiation showed no differences between healthy and DM1 cells. However, after 7 days in the differentiation medium, OA levels in DM1 cells dropped by half compared to controls. Interestingly, differences in OA levels between control and DM1 were higher in fibroblast-derived cells compared to myoblasts. Additionally, in all cases OA levels correlated with SCD1 reduction in these cell types. Moreover, it is worth mentioning that SCD1 is a short-lived multispanning ER membrane protein reported to be ubiquitously degraded by the ubiquitin-proteasome system (UPS). An abnormal increase in UPS activity was described in skeletal and cardiac muscle from DM300 and DMSXL model mice [61, 62]. Consistent with these observations, studies in human myoblasts from DM1 patients confirmed abnormal overexpression of UPS-related genes [35]. It was also demonstrated that miR-7 regulates UPS in these cells as supplementation of DM1 muscle cells with a miR-7 mimic repressed UPS while treatment of control cells with an antagomiR promoted UPS-related genes expression [16]. Thus, considering our results, the overactivated UPS system may promote the degradation of SCD1 over its synthesis leading to fatty acid dysregulation. In addition, OA supplementation enhances miR-7 biogenesis, which impacts UPS by inhibiting its activity. All this may translate into a decrease in SCD1 degradation through the UPS pathway, generating a regulatory loop that improves the alterations generated by the DM1 pathological conditions (Fig. 8). DM1 is classified as a spliceopathy, and it is reasonable to speculate that besides a reduced expression of SCD1, the enzyme has lower activity due to defective alternative splicing regulation. However, this hypothesis is invalidated by the fact that only one isoform of *SCD1* has been described [63]. Interestingly, several independent studies reported a very tight and complex regulation of SCD1 gene expression in response to hormonal factors. DM1-related changes in insulin signaling have been reported in ∼30 clinical studies, and insulin resistance is a key phenotype [64] due to, at least partially, the aberrant regulation of *insulin receptor* (*INSR*) splicing [65]. In addition, serum leptin and glucagon levels in DM1 patients are pathologically increased [66, 67] Specifically, insulin is a potent activator of SCD1 transcription [68] while leptin was reported to inhibit SCD1 expression [69]. Additionally, regulation of SCD1 expression by leptin is of particular interest because in vivo the role of insulin seems to be subordinate to that of leptin, thus suggesting a crosstalk between the two signaling pathways [70]. Similarly, glucagon has also been described as an inhibitor of SCD1 transcription, possibly due to a direct inhibition of the insulin-stimulating effects [71]. Deficient insulin signaling contributes to mTOR/AKT downregulation in DM1, which may fail to activate *sterol regulatory element binding protein 1c* (*SREBP-1 C*), a direct transcriptional regulator of fatty acids synthesis genes and dependent of [72–74] This hypothesis is supported by our results, where we observed that adding an LXR agonist to DM1 cells is sufficient to restore OA levels. Globally, it is likely that defects in the aberrant splicing of *INSR* contribute to SCD1 downregulation in DM1. Moreover, it cannot be ruled out that the induction of *SCD1* expression is the only reason that rescues OA levels, since LXR is not specific to SCD1 but activates other genes, including those involved in the cholesterol metabolism such as *ABCA1* or *RNF145*, an integral cell membrane protein that exports of excess cholesterol from cells [54, 75]. *ABCA1* expression has been shown to respond to OA levels themselves [76], raising the possibility that the strong increase in *ABCA1* levels may stem from a direct effect by LXR and indirectly by the rise in OA in comparison to the more modest increases in expression for *SCD1* and *RNF15* whose transcription is also regulated by LXR.

OA supplementation has been shown to promote myoblast cell proliferation and differentiation in vitro [32, 33, 77]. DM1 myotubes have strongly altered these parameters. However, we observed a complete reversal of differentiation after OA treatment of the cells. This pronounced effect may be due to the sum of the effect of OA on cell differentiation and the MSI2 > miR-7 axis, whose modulation by different strategies we have previously observed results in significant improvements in cell differentiation-related parameters [16, 21]. As determined by DESMIN immunodetection, it is interesting to note that OA treatment led to an increase in myotube fusion capacity and myotube diameter comparable to values previously obtained when MSI2 was silenced by gapmer antisense oligonucleotides or inhibited by the small molecule Ro 08-2750 [21]. However, in previous work, we reported no effect on the diameter upon miR-7 supplementation by mimetics [16]. These results suggest that while there is a clear link in DM1 pathology between MSI2 and miR-7, MSI2 may also contribute to pathology independently of miR-7. As shown, MSI2 down-regulates P21, which is a well-known inhibitor of the cell cycle and can arrest its progression in G1/S and G2/M transitions by inhibiting CDK4,6/cyclin-D and CDK2/cyclin-E, respectively [78]. At this point, it is important to highlight the relationship between levels OA and P21, likely mediated by the activity of MSI2. Specifically, in TDM, we observed that treatment with OA increased P21 to levels similar to those observed in control cells. However, in immortalized myoblasts, although significant, the increase in P21 did not reach the levels observed in healthy cells. These findings are consistent with the results obtained for the fusion index achieved by cells upon supplementation with OA. In aggregate, these findings suggest a correlation between OA levels, P21 expression, and cellular differentiation outcomes. Thus, it is possible that de-repression of P21 after OA treatment is sufficient to enhance terminal differentiation of myotubes thus promoting an increase in myotubes size.

Cell membrane integrity has been studied in different murine models and DM1 patient biopsies. The authors concluded that the membranes in skeletal muscle, heart, and brain from DM1 tissue do not exhibit compromised membrane integrity [79]. In animals, cell membranes consist of various lipid species, classified into three main classes: glycerophospholipids, sphingolipids, and cholesterol. The presence of OA in the phospholipids of the cell membrane can regulate its structure and modify its biophysical properties. Additionally, OA plays a role in stabilizing the bilayer structure and regulating the proteins embedded within the membrane [80]. It is possible that the OA deficit observed in DM1, which may be partly attributed to a defect in its biosynthesis, is not sufficient to negatively impact the structure or activity of affected individuals’ cell membranes. However, considering that the presence of OA in the organism is derived from both endogenous biosynthesis and dietary intake [81], it is possible that the dietary source is enough to maintain membrane integrity while not adequately interacting with MSI2. Further research is needed to explore the precise mechanisms underlying these observations and their implications in DM1.

## Conclusion

Fatty acid metabolism was previously unknown to contribute to muscle differentiation defects in DM1. This study demonstrates reduced levels of OA in three patient-derived cell lines and reveals the potential of OA supplementation for treating DM1. While the OA deficit synergizes with the previously known inhibition of MSI2 on miR-7 biogenesis, OA supplementation inhibits excessive MSI2-mediated repression on miR-7 levels contributing to lower catabolism by autophagy and UPS systems, enhanced expression of cell cycle progression inhibitor P21, and rescue of muscle cell phenotypes. Effects on cell differentiation in vitro were specific to OA since isoschizomer EA could not modulate MSI2 activity on RNA targets nor rescue disease-associated cell defects. Our data also show that the critical OA synthesis enzyme SCD1 is reduced in DM1 cells, likely contributing to the OA deficit.

## Electronic supplementary material

Below is the link to the electronic supplementary material.

Supplementary Material 1

## Data Availability

This study includes no data deposited in external repositories. All data are available in the article and/or additional files and via corresponding authors upon reasonable request.
