# Supplementary material for: Therapeutic potential of oleic acid supplementation in myotonic dystrophy muscle cell models
Source: Biol Res. 2024 May 17;57:29. doi: 10.1186/s40659-024-00496-z (PMC11100173; doi:10.1186/s40659-024-00496-z)
Supplement: Supplementary file 1 — Supplementary Material 1 [file 40659_2024_496_MOESM1_ESM.pdf]

Supplementary Information

Therapeutic potential of oleic acid supplementation in myotonic dystrophy muscle cell models

Nerea Moreno, Maria Sabater-Arcis, Teresa Sevilla, Manuel Perez Alonso, Jessica Ohana, Ariadna Bargiela*, and Ruben Artero

*corresponding author

E-mail: ariadna_bargiela@iislafe.es

This PDF file includes:

Supplementary Figures 1 to 8

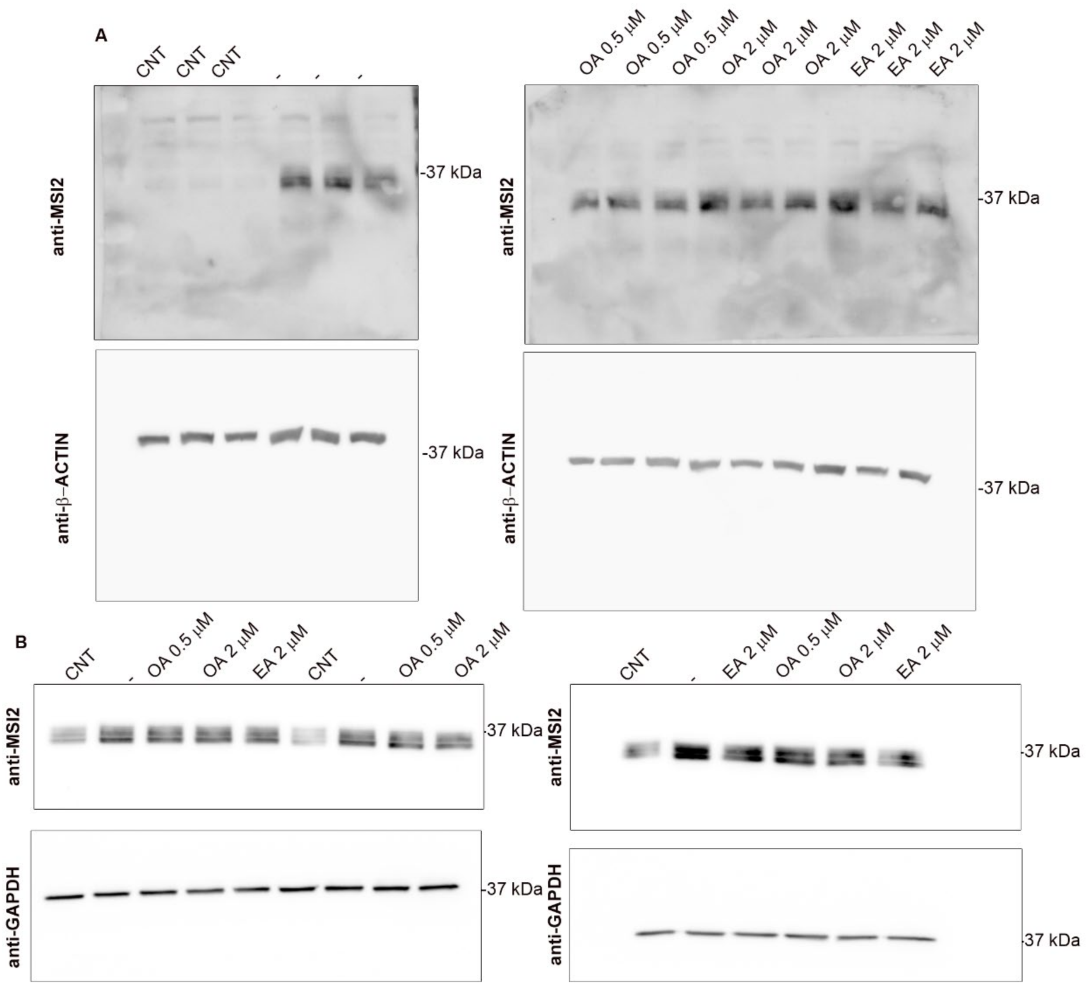

Supplementary Figure 1 Uncropped western blots for Fig. 2C and 6B (A) and Fig. 2E and 6D (B)

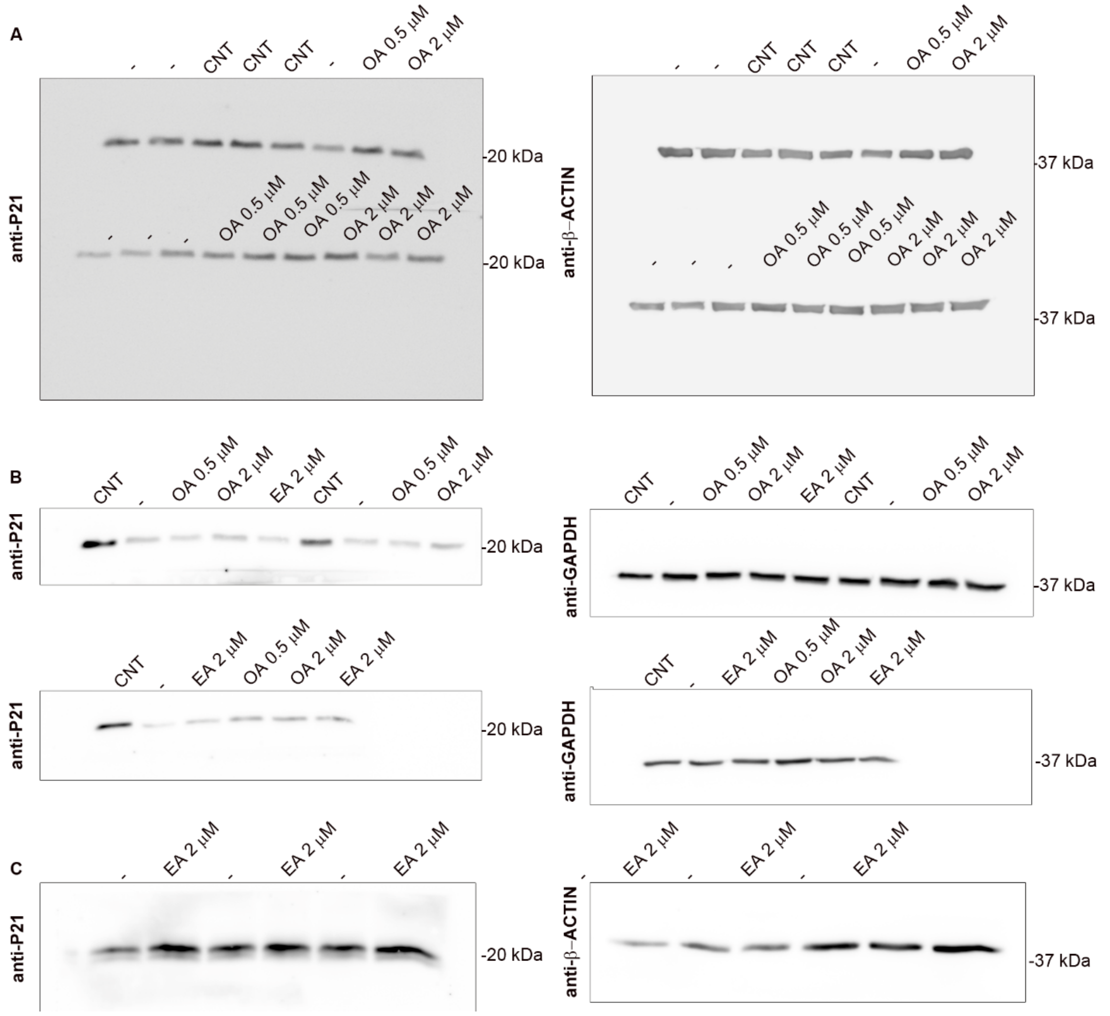

Supplementary Figure 2 Uncropped western blots for Fig. 2H (A), Fig. 2J and 6I (B) and Fig. 6G (C)

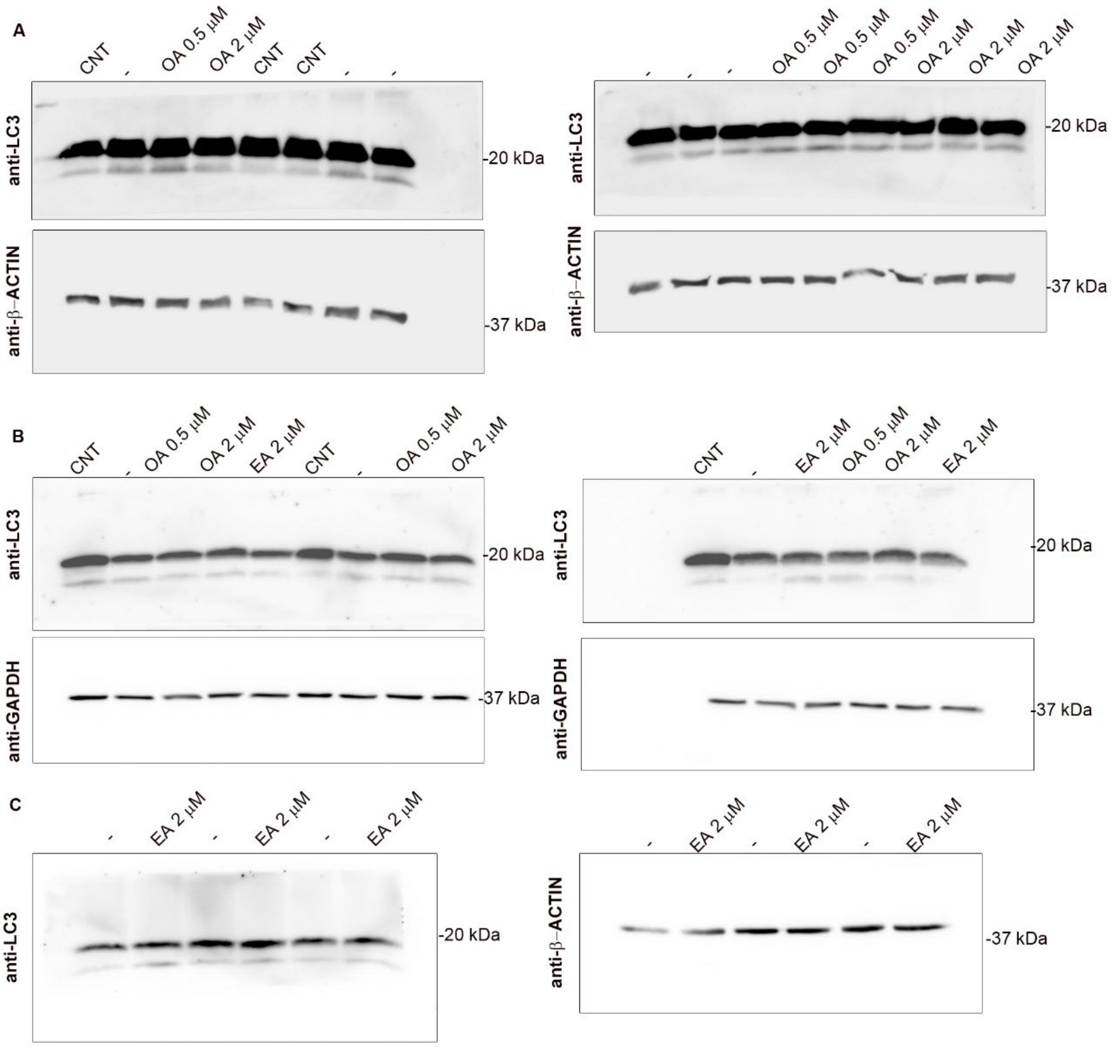

Supplementary Figure 3 Uncropped western blots for Fig. 3T (A), Fig. 3V and 6M (B) and Fig. 6K (C)

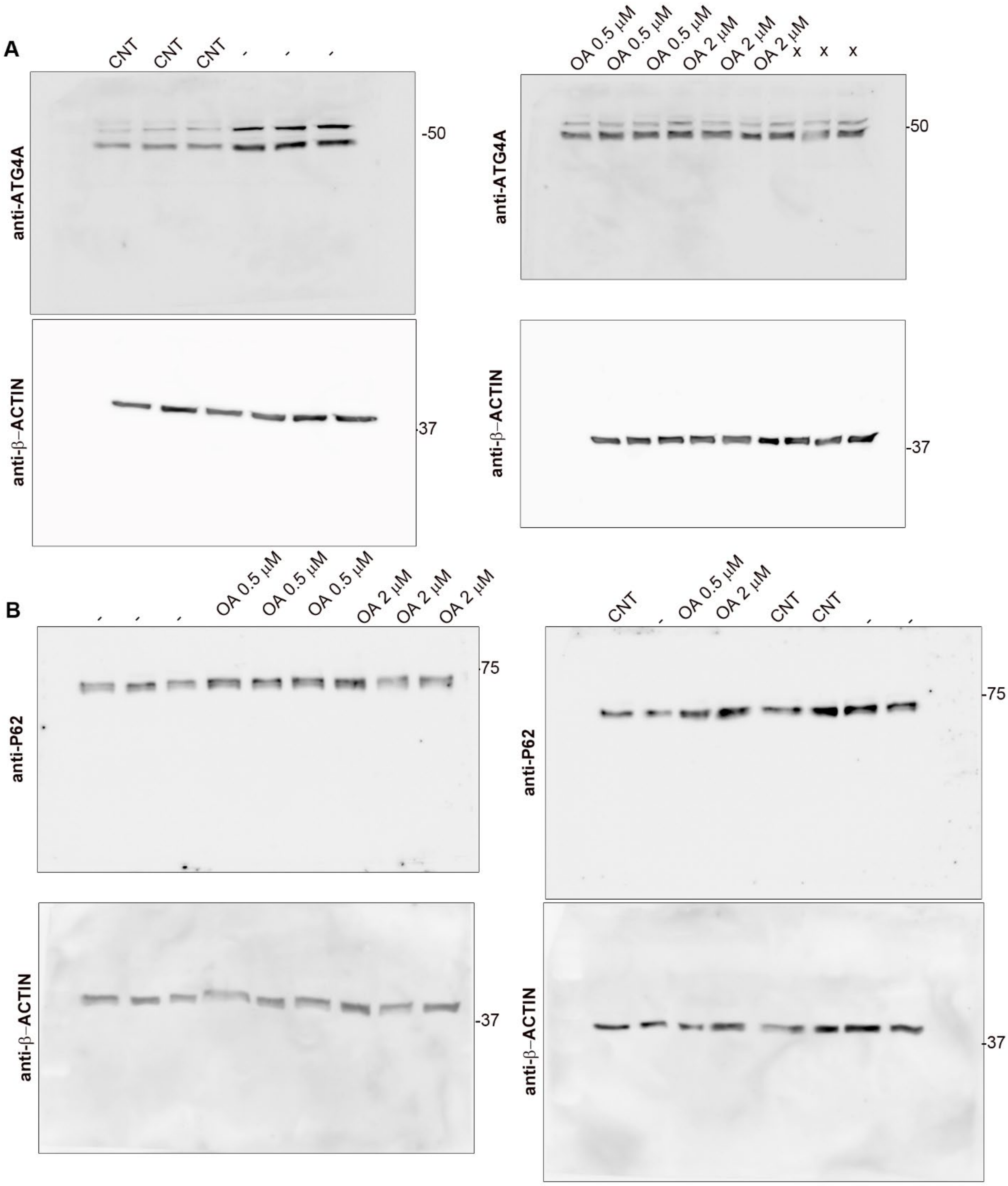

Supplementary Figure 4 Uncropped western blots for Fig. 3U (A) and Fig. 3V (B). Crosses mark membrane lanes from unrelated experiments

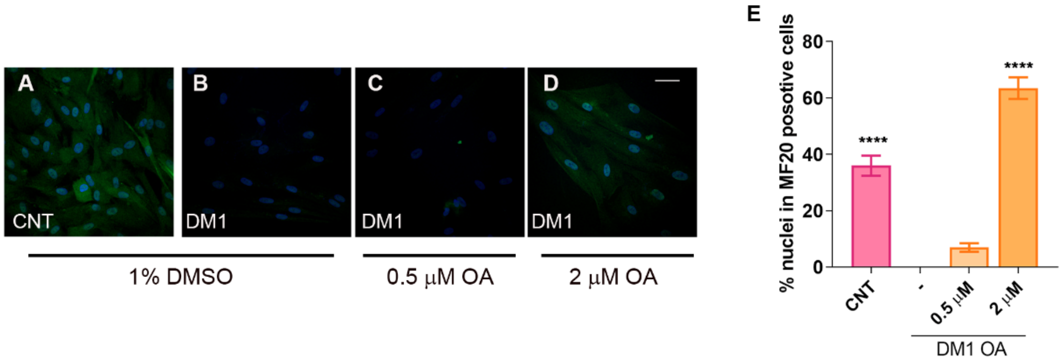

Supplementary Figure 5 MF20 immunostaining in OA-treated TDMs (A-D) Representative confocal micrographs of MF20 immunostaining (green) in 7-day-differentiated control or DM1 TDMs treated with the indicated compounds and concentrations. (E) Quantification of the percentage of nuclei within MF20 positive cells in each condition. Nuclei were stained with DAPI. Scale bar: 40 µm. All data were compared to cells treated with DMSO (-). The bar graphs show the mean±SEM; P < 0.0001, according to the one-way ANOVA test.

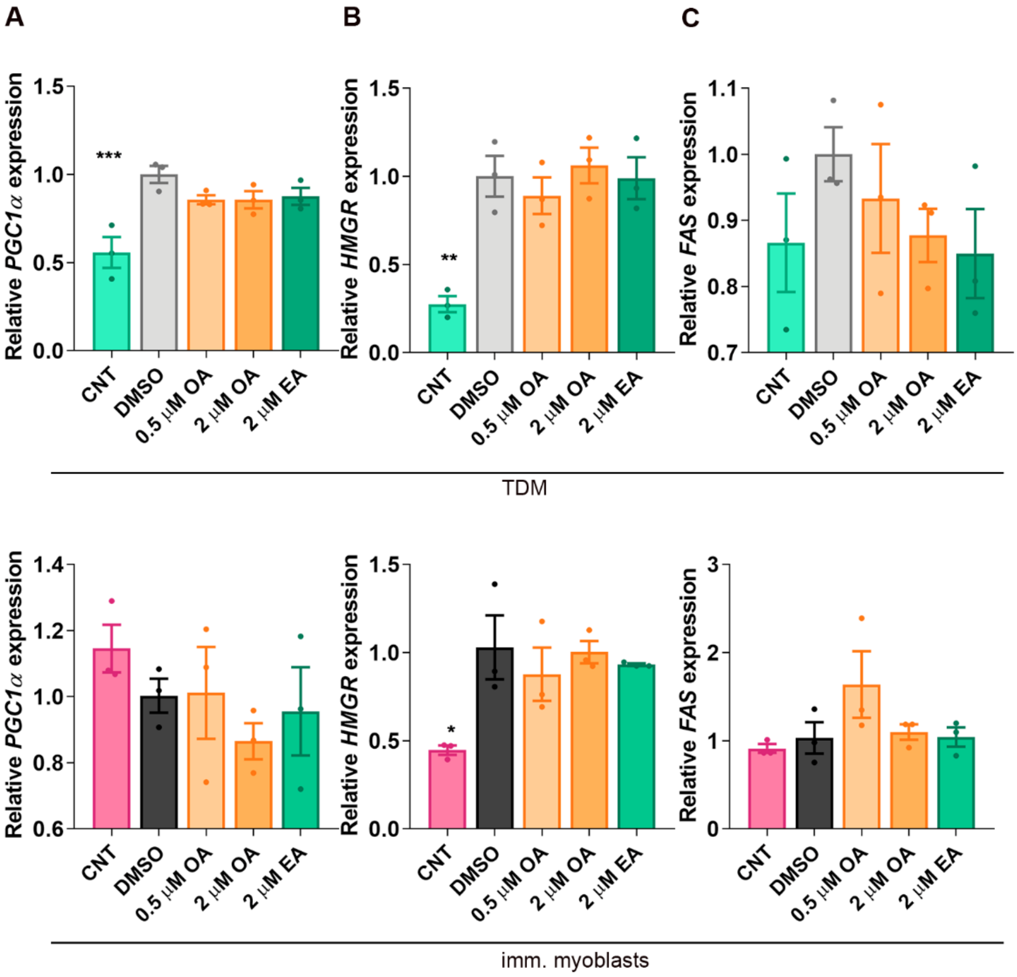

Supplementary Figure 6 Quantification by RT-qPCR of the relative expression of (A) *PCG1α*, (B) *HMGR*, and (C) *FAS* in control and DM1 TDM (upper panels) or immortalized myoblasts (lower panels) treated with DMSO as vehicle or with the indicated concentrations of OA or EA. Gene expression was referenced to *GAPDH,* and *GPI* expression. At least three independent experiments were carried out, and three technical replicates were performed from each

biological sample. The bar graphs show the mean ± SEM *P<0.05, **P<0.01, *** P<0.001 according to one-way ANOVA test.

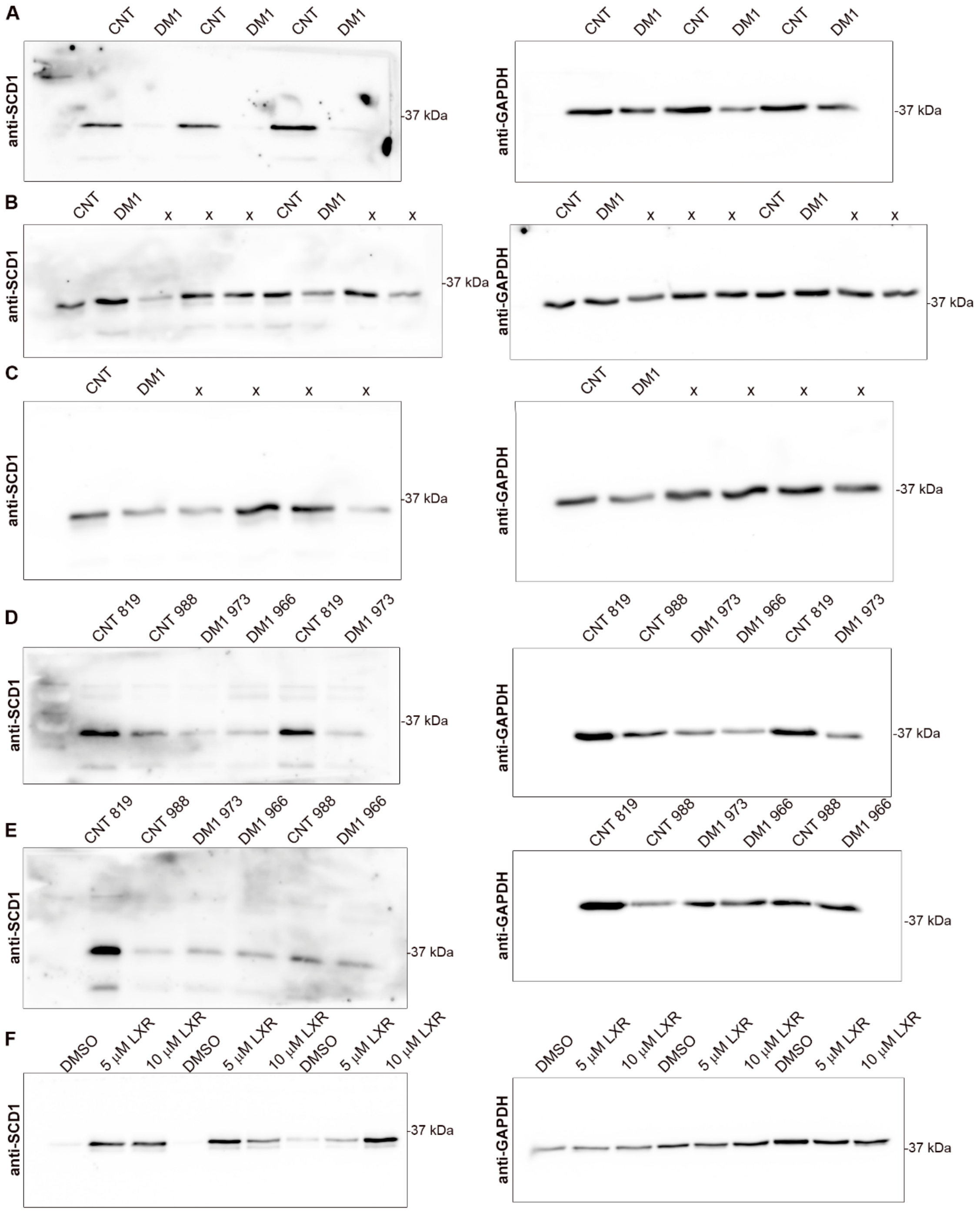

Supplementary Figure 7 Uncropped western blots for Fig. 7B (A), Fig. 7D (B,C), Fig. 7F (D,E) and, Fig. 7J (F)

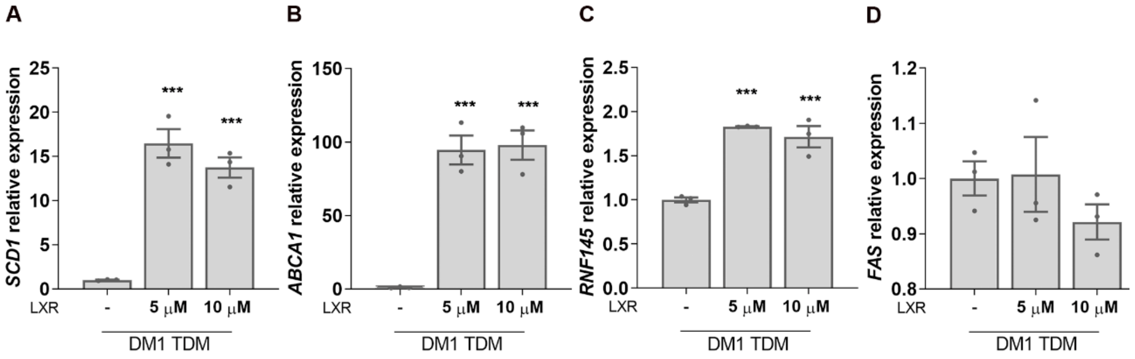

Supplementary Figure 8 Quantification by RT-qPCR of the relative expression of (A) *SCD1*, (B) *ABCA1*, (C) *RNF15*, and (D) *FAS* in DM1 TDM treated with the indicated concentrations of LXR agonist. Gene expression was normalized to *GAPDH,* and *GPI* expression. Three independent experiments with three technical replicates from each biological sample were performed. The bar graphs show the mean ± SEM *** P<0.001 according to one-way ANOVA test.
